# When Thyrotoxicosis Meets a Latent Plaque: Unmasking a Type I Myocardial Infarction

**DOI:** 10.7759/cureus.102216

**Published:** 2026-01-24

**Authors:** Joseph Muñoz, Damini Kashyap, Aleksandr Kouk, Thomas Greco, Joseph Maslak

**Affiliations:** 1 Internal Medicine, HCA Citrus Hospital, Inverness, USA

**Keywords:** acute coronary syndrome, nstemi type 1, plaque rupture, subclinical atherosclerosis, thyrotoxicosis

## Abstract

Thyrotoxicosis is a rare but significant precipitant of acute coronary syndrome (ACS), especially in young individuals without traditional cardiovascular risk factors. We present the case of a 36-year-old male with no past medical history who arrived with substernal chest pain, elevated troponins, and sinus rhythm without ST changes on ECG. Thyroid ultrasound revealed a large goiter, and labs confirmed severe thyrotoxicosis. Coronary angiography showed 85% stenosis of the proximal-to-mid left anterior descending artery, managed successfully with stent placement. This case demonstrates how an excess of thyroid hormone may contribute to the development of type I myocardial infarction. Thyrotoxicosis should be included in the differential diagnosis of ACS in young patients without traditional risk profiles to ensure timely diagnosis and targeted therapy.

## Introduction

Acute coronary syndromes (ACS) are defined as a sudden reduction in coronary blood flow to the heart [1]. They are classified as spectrum diseases, including ST-segment elevation myocardial infarction (STEMI), non-ST-segment elevation myocardial infarction (NSTEMI), and unstable angina (UA). The ACS classifications depend on the patient's presenting symptoms, electrocardiogram findings, and biomarker profiles. UA presents with ischemic chest pain but lacks elevation of cardiac biomarkers and persistent ECG changes; NSTEMI shows elevated troponins signifying myocardial injury, yet without ST-segment elevation; STEMI is characterized by both elevated biomarkers and persistent ST-segment elevation, indicating full-thickness myocardial infarction [1,2]. Prompt risk assessment facilitates the decision on whether to consider early intervention or conservative management to reduce mortality. Essential management includes early percutaneous coronary intervention when indicated, dual antiplatelet therapy, and anticoagulation as primary management, as well as statins, beta blockers, and renin-angiotensin system inhibitors as secondary management [1-3]. Thyrotoxicosis most commonly precipitates type II myocardial infarction or demand ischemia, with type I myocardial infarction being rare. Excess circulating thyroid hormones increase myocardial oxygen demand, heart rate, and contractility, and sensitize adrenergic receptors, creating a hyperdynamic state that can precipitate coronary vasospasm, endothelial dysfunction, and even myocardial infarction in the absence of obstructive coronary artery disease [4-6]. In this case, the patient presented with thyrotoxicosis and was found to have an NSTEMI secondary to obstructive coronary artery disease.

## Case presentation

**Figure 1:**
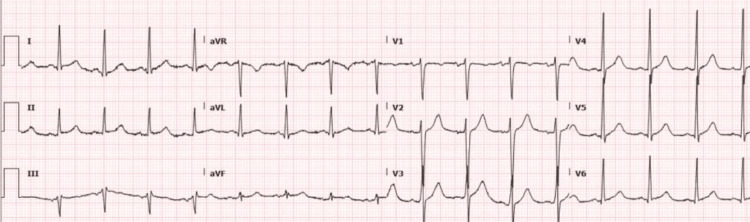
Electrocardiography demonstrating sinus rhythm without ST changes

**Figure 2:**
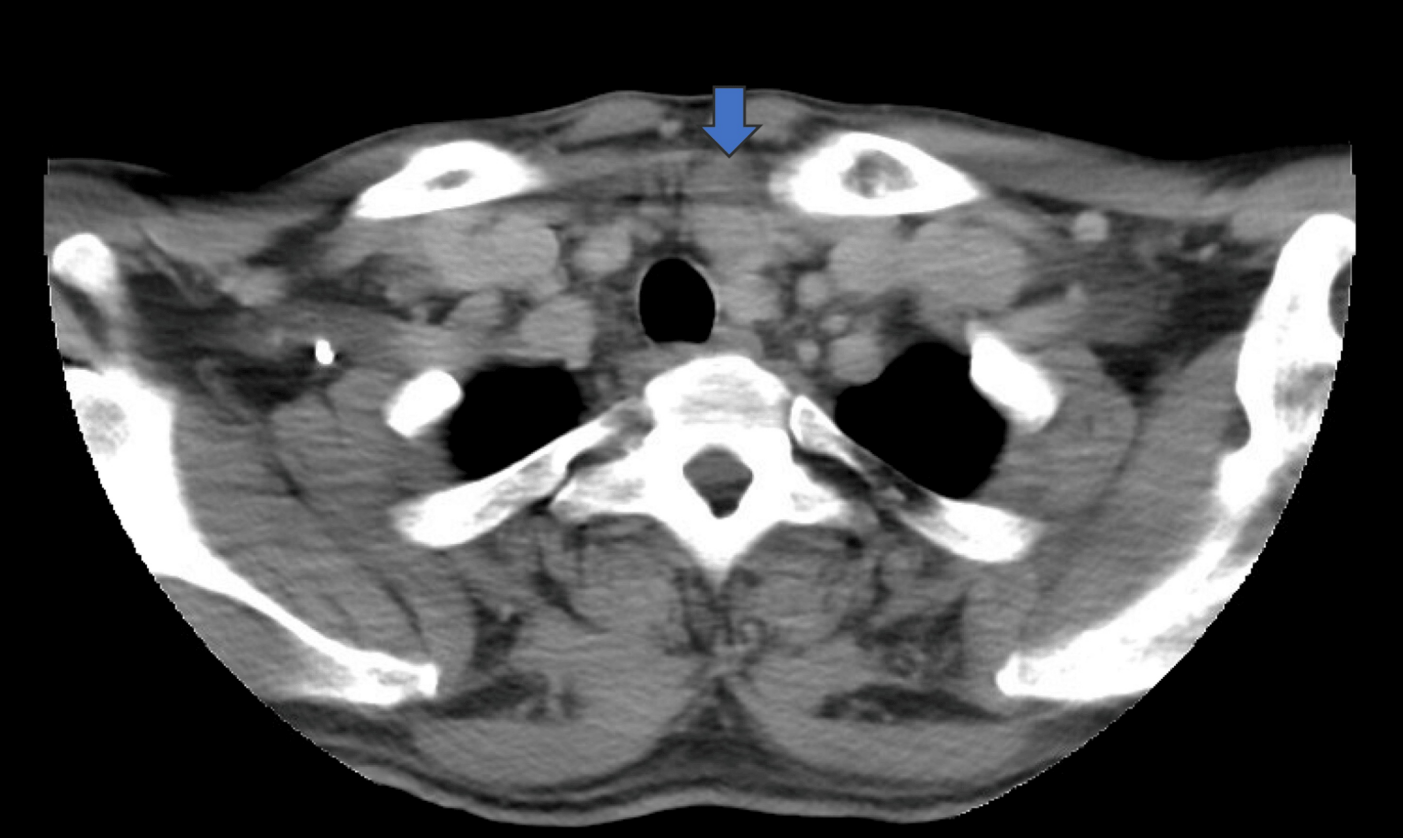
CT of the neck showed a massive goiter with a 2.1 cm exophytic nodule at the lower pole of the left thyroid lobe

**Figure 3:**
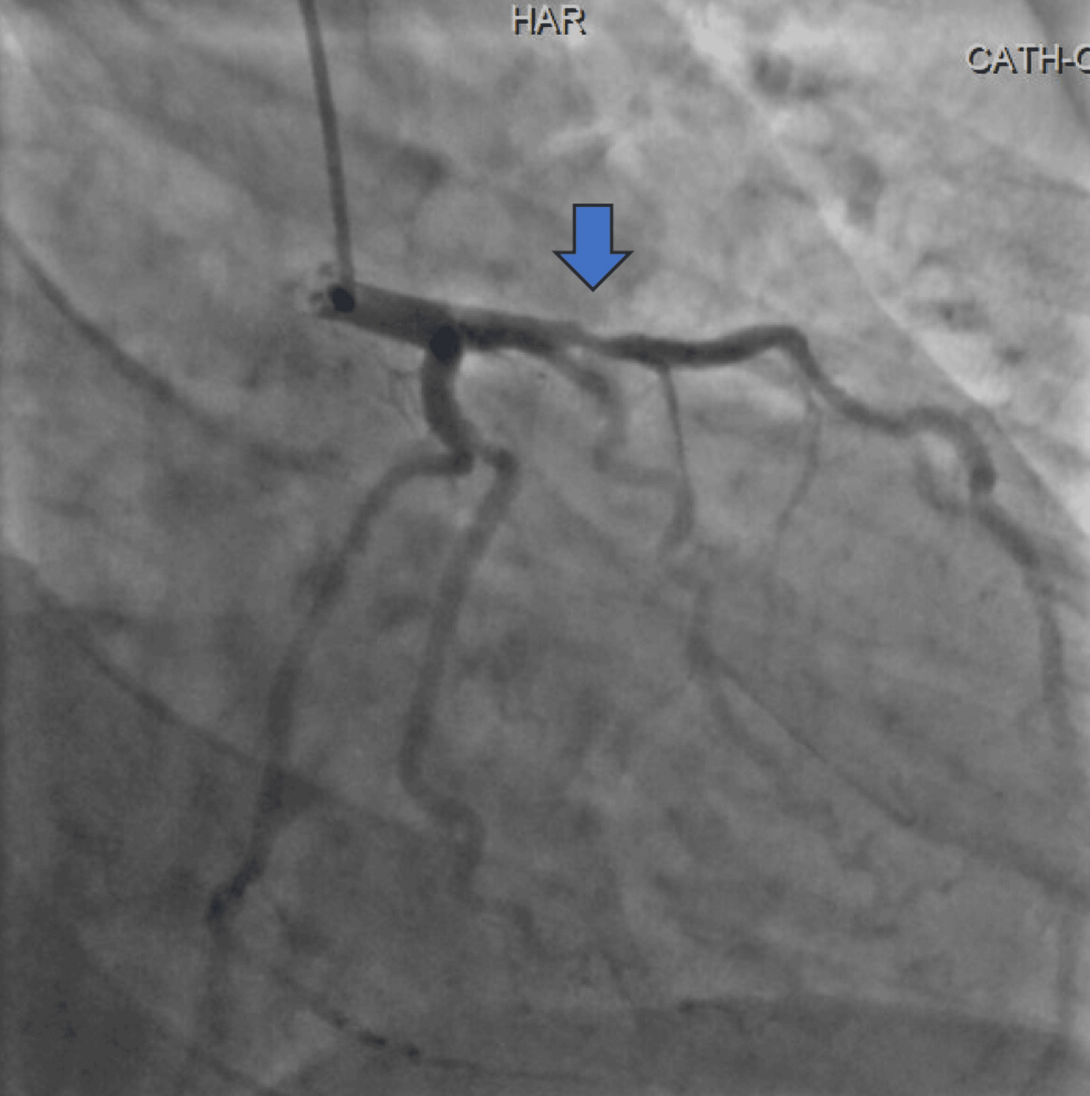
Left heart catheterization demonstrating 85% de novo stenosis in the proximal to mid left anterior descending artery (12 mm length)

**Figure 4:**
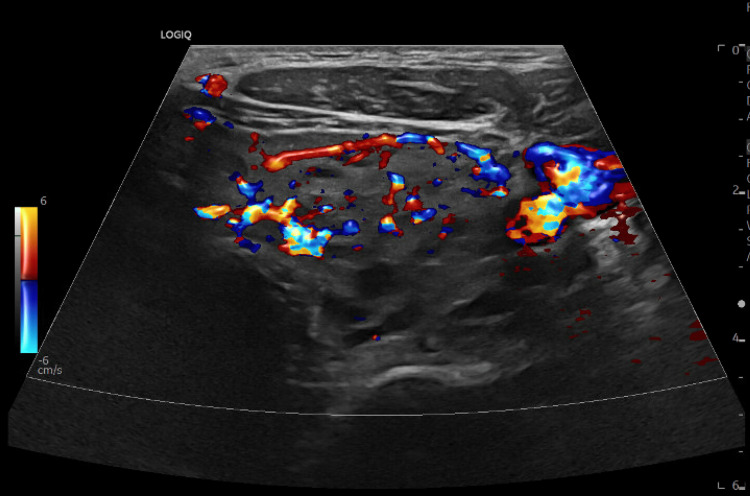
Ultrasound of the thyroid showing a diffusely heterogeneous thyroid with increased vascularity

A 36-year-old male presented to the emergency department with substernal chest pain radiating to the left hand. The patient did not have any history of hypertension, diabetes mellitus, tobacco use, or hyperlipidemia. He reported an unintentional weight loss of 80 pounds over the past six months, despite maintaining a good appetite. His father had undergone a partial thyroidectomy for an unspecified thyroid condition. Upon arrival, the patient's chest pain improved after application of a nitroglycerin patch. His systolic blood pressure (SBP) was in the 140s mmHg; all other vital signs were within normal limits. Laboratory studies revealed a thyroid-stimulating hormone (TSH) level of < 0.020 µIU/mL, free T4 of 3.5 ng/dL, free T3 > 22.8 pg/mL, anti-thyroid peroxidase antibody of 237 IU/mL, anti-thyroglobulin antibody of 42.4 IU/mL, and elevated troponin I of 791.2 ng/L, with a subsequent value of 695.8 ng/L (Table 1). Electrocardiography demonstrated sinus rhythm without ST changes (Figure 1). CT of the neck showed a massive goiter with a 2.1 cm exophytic nodule at the lower pole of the left thyroid lobe (Figure 2). Cardiology consultation raised concern for NSTEMI. Transthoracic echocardiography showed a normal valve function with an ejection fraction of 55-60%. Left heart catheterization revealed an 85% de novo stenosis in the proximal to mid left anterior descending artery (12 mm length), which was successfully treated with stent placement (Figure 3). Given the suppressed TSH and goiter on imaging, a dedicated thyroid ultrasound and panel were obtained before coronary angiography. Ultrasound showed a diffusely heterogeneous thyroid with increased vascularity (Figure 4). The differential diagnoses comprised toxic multinodular goiter, Graves disease, and neoplastic thyroid disease. He was discharged the day after percutaneous coronary intervention on aspirin 81 mg daily, atorvastatin 40 mg daily, isosorbide mononitrate 30 mg daily, metoprolol tartrate 25 mg twice daily, and prasugrel 10 mg daily. He was advised to follow up with cardiology for medication management and to establish care with endocrinology for further evaluation of his thyroid pathology.

**Table 1:** Summary of thyroid and cardiac biomarkers TSH: thyroid-stimulating hormone

Test	Results (Initial)	Results (Follow-up)	Units	Reference Range
TSH	<0.020	-	µIU/mL	0.45-4.50
Troponin I	791.2	695.8	ng/L	<34
Free T3	>22.8	-	pg/mL	2.0-4.4
Free T4	3.5	-	ng/dL	0.8-1.8
Anti-TPO Ab	237	-	IU/mL	<35
Anti-TB Ab	42.4	-	IU/mL	<40

## Discussion

ACS is rare in young, low-risk adults [1,2]. Cardiac events occur 2.6 times more frequently when thyroid levels are increased [3]. Our case highlights an unconventional presentation in which the patient was diagnosed with type I myocardial infarction in the setting of thyrotoxicosis, with coronary angiography revealing a stenosis of 85% in the LAD, requiring stent placement. Common risk factors for patients developing acute coronary syndromes include smokers, diabetics, hypertensives, and hyperlipidemic patients [1]. The absence of conventional factors in our patient increases the likelihood of considering differentials. The vast majority of cases of thyrotoxicosis are associated with type II myocardial infarction [5,6]. This type is characterized by either high oxygen demand or decreased supply, eg, severe sepsis, anemia, or arrhythmias. Cardiac catheterization typically reveals non-obstructive vessel disease or minor, stable plaques [1]. In contrast, a type I myocardial infarction is an acute atherothrombotic event in which a plaque ruptures, leading to thrombus formation [4,7]. This can cause partial or complete obstruction of the vessel, reducing blood flow to the coronary arteries. Electrocardiograms can show both ST-segment elevation and depression [2]. Thyroid hormones upregulate beta-adrenergic receptors, amplifying catecholamine responsiveness [5]. The result is increased heart rate (positive chronotropy), contractility (positive inotropy), and myocardial oxygen consumption, potentially leading to demand ischemia. In thyrotoxicosis, even moderate coronary stenosis can be insufficient to meet elevated demands, precipitating ACS [6]. A chronic hyperthyroid state induces hyperdynamic circulation, leading to left ventricular hypertrophy. This can cause reduced diastolic filling time and elevate myocardial wall stress, which may accelerate ischemic changes [5]. Additionally, high levels of T3 promote nitric oxide synthesis, predisposing to alterations in vascular tone and increasing susceptibility to vasospasm. Thyrotoxicosis can also disrupt endothelial barrier function, fostering local inflammation and plaque destabilization [8]. Chronic hyperthyroidism can lead to changes in the endothelial lining and a plaque-promoting state. Although hyperthyroidism has been associated with decreased high-density lipoprotein (HDL), increased low-density lipoprotein (LDL), and oxidized lipids, these findings were not observed in our patient. His lipid panel was within normal limits. Additionally, enhanced platelet aggregation can contribute to the prothrombotic state [6,9]. Our patient had a normal BMI and lipids and did not have any visible risk factors. Subclinical atherosclerosis could explain the 70% left anterior descending (LAD) stenosis, but it is difficult to make a direct association in a single case. Subclinical atherosclerosis is characterized by the early formation of plaques in the vascular system, without any clinical signs [1]. The 85% LAD stenosis found in our patient may reflect accelerated subclinical atherosclerosis, now unmasked due to increased myocardial demand under a thyrotoxic state. This case supports the rationale that excess thyroid hormone can silently drive vascular pathology, culminating in ACS [3,6]. This non-traditional presentation underscores the importance of considering a broad range of possibilities when a young adult presents to the emergency department with symptoms suggestive of possible ACS [2,10]. Our patient's clinical presentation on arrival included chest pain radiating to the left arm with elevated levels of troponins, which is often seen in ACS. However, his age, combined with his lack of risk factors, warrants consideration of alternative or compounding etiologies. The discovery of profound thyrotoxicosis, supported by suppressed TSH, elevated T3/T4, and thyroid autoantibodies, reveals a critical but often overlooked contributor to acute coronary pathology [5,6].

## Conclusions

This case emphasizes the importance of considering a broad range of potential diagnoses when a young adult presents to the emergency department with symptoms suggestive of ACS. Thyrotoxicosis should be recognized as a potential precipitant in ACS among young, low-risk patients and should undergo further cardiovascular evaluation. Rapid identification and a coordinated multidisciplinary approach are essential for optimizing outcomes and preventing recurrence.
